# Pannexin-1 hemichannels promote experimental colitis inflammation in a CD4^+^ T cell-specific manner

**DOI:** 10.3389/fimmu.2025.1621353

**Published:** 2025-10-08

**Authors:** Pooja Rani Mina, Bruna de Gois Macedo, Caio Loureiro Salgado, Chloe Liliana Leff, Daniel Bihnam, Henrique Borges da Silva

**Affiliations:** ^1^ Department of Immunology, Mayo Clinic, Phoenix, AZ, United States; ^2^ Department of Cancer Biology, Mayo Clinic, Phoenix, AZ, United States

**Keywords:** CD4^+^ T cells, Th2 (type-2) immune responses, colitis, pannexin 1 (Panx1), neutrophils

## Abstract

**Background:**

The development of ulcerative colitis (UC) is associated with inflammatory responses driven by effector CD4^+^T cells, including type 3 (Th17) cells and atypical pathogenic type 2 (Th2-like) cells. UC is also linked to accumulation of neutrophils that can amplify intestinal damage. The mechanisms behind the accumulation of colitogenic CD4^+^ T cells are not fully understood, particularly regarding how regulators of intracellular versus extracellular metabolites can drive such responses.

**Main findings:**

Here, we found that Pannexin-1 (PANX1) hemichannels, which promote ATP export to the extracellular environment, are crucial for the development of colitis. We found that PANX1, which is upregulated in UC patients, is required for the induction of colitis in multiple experimental models. The role of PANX1 is effector T cell-specific and is correlated with the accumulation of TNF-α producing pathogenic Th2-like cells. Effector conversion of CD4^+^ T cells into Th2-like cells depends on PANX1. Finally, PANX1-mediated pathogenic CD4^+^ T cell responses correlate with the accumulation of neutrophils during colitis.

**Conclusions:**

Together, our results suggest that PANX1 promotes colitis-associated pathogenic Th2-like responses and a possible link between these cells and colitis neutrophilia.

## Introduction

Inflammatory bowel diseases such as ulcerative colitis (UC) are triggered by a complex network of genetic, environmental and immunological components, impacting >5 million patients worldwide (1). The development of pathogenic T cell responses significantly drives the progression of UC, which has prompted intense investigation about the nature of these T cell responses (2). Pathogenic CD4^+^ T cells are strongly associated with the progressive development of UC in patients (3, 4) and are important targets of current and future therapies aiming to block or revert UC progression (5, 6). Multiple subsets of effector CD4^+^ T cells can promote distinct aspects of UC inflammation (3). Among these, the role of type 17 (Th17) CD4^+^ T cells is relatively well established (3, 7). In contrast, much less is known about how type 2 (Th2)-like CD4^+^ T cells can cause pathogenic responses and promote UC (8).

Effector differentiation of CD4^+^ T cells occur through a series of alterations at the transcriptional, epigenetic and metabolic levels (9). Transcriptional regulation of effector CD4^+^ T cell fate is relatively well-studied, highlighting for example the importance of RORγT and GATA3 for the differentiation of Th17 and Th2 cells, respectively (9). In contrast, the metabolic regulators of their function are less known. Among the ways by which CD4^+^ T cells can metabolically control their function and homeostasis, the expression of membrane-bound transporters is crucial for the control of intracellular versus extracellular metabolite levels (10–12). Many of these transporters are metabolite importers, such as membrane-bound ion channels which lead to intracellular increases in Ca^2+^, for example (13). In contrast, metabolite exporters can control metabolic pathways through both induction of extracellular metabolite accumulation and negative regulation of intracellular metabolite levels. Among these exporters, Pannexin-1 (PANX1) channels can negatively regulate pathogenic Th2 cell responses in the context of lung allergens through accumulation of extracellular ATP that is promptly converted into immunosuppressive adenosine (14). PANX1 is constitutively expressed in all T cells and reportedly increases in expression upon T cell priming (14, 15). It is possible, therefore, that PANX1 expression could play a role in Th17 or Th2-like responses against UC. Recent reports suggest a role for PANX1 in experimental colitis (16, 17), although no clear link to CD4^+^ T cell responses was studied.

In this report, we used mouse models to demonstrate that PANX1 promotes experimental colitis development in a CD4^+^ T cell-dependent way. We found that PANX1-knockout (PANX1-KO) mice do not develop Dextran Sodium Sulfate (DSS) colitis, and PANX1-KO CD4^+^ T cells fail to induce experimental colitis in Rag2-KO mice. Colon-infiltrating PANX1-KO CD4^+^ T cells had decreased numbers of GATA-3-expressing cells but consistent numbers of Th17 cells. These Th2-like cells co-expressed type 2 cytokines and TNF-α, suggesting a pathogenic phenotype present in the wild type mice but absent in the PANX1-KO mice. The PANX1 role in pathogenic Th2-like cell formation has a cell-intrinsic component, since PANX1-KO CD4^+^ T cells are defective in developing into TNF-producing Th2 cells *in vitro*. Finally, the defects in pathogenic Th2-like cells in colitis-exposed PANX1-KO mice are associated with decreased neutrophilia, suggesting a possible link between these two colitogenic immune aspects.

## Results

### Expression of PANX1 by CD4^+^ T cells promote the development of experimental colitis

**Figure 1:**
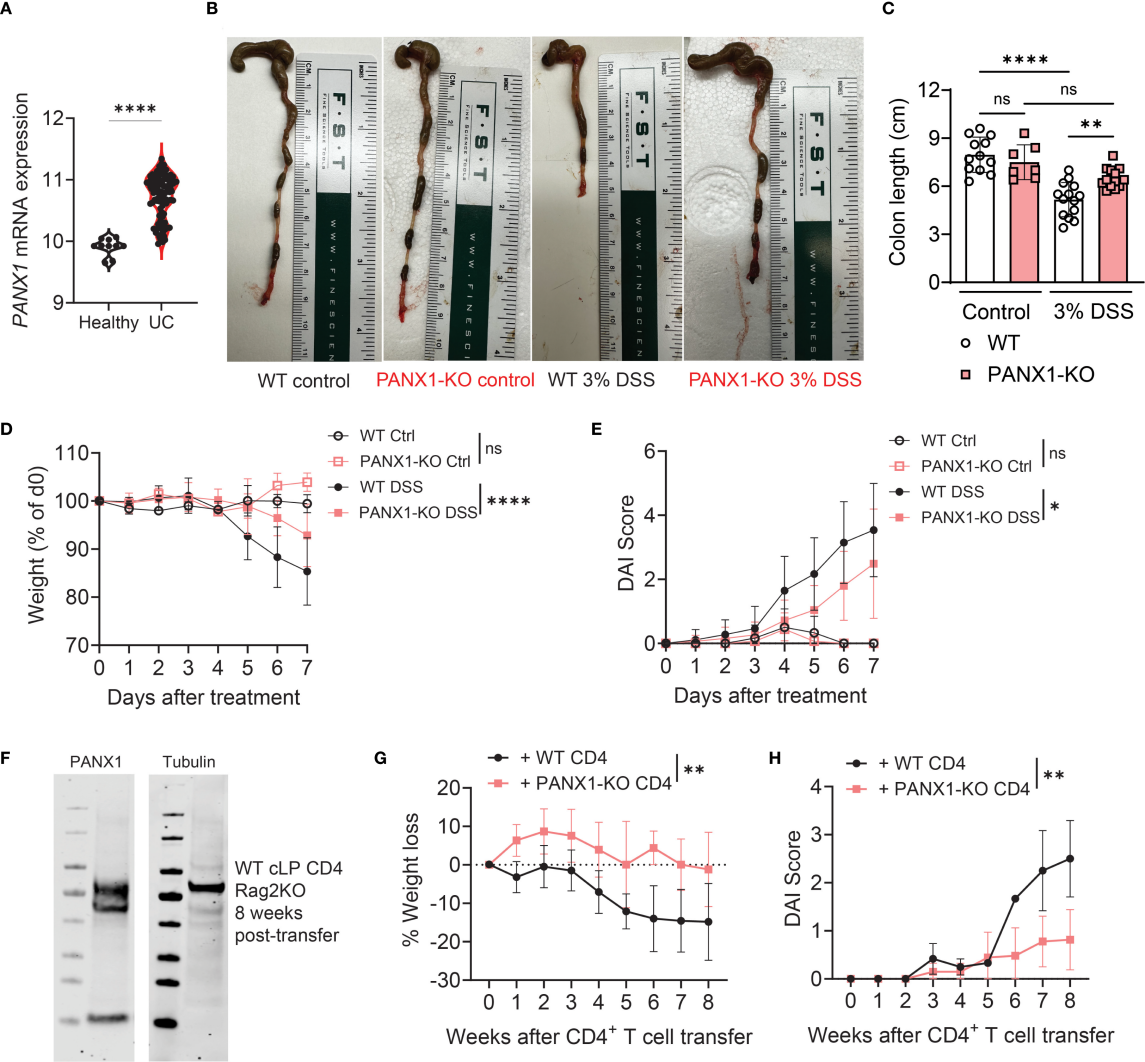
CD4^+^ T cell expression of PANX1 promotes the development of experimental colitis. **(A)** Human average *PANX1* mRNA expression in the large intestines of healthy subjects (Healthy) versus ulcerative colitis patients (UC). **(B–E)** WT and PANX1-KO mice were treated between days 0–7 with PBS (control) or 3% Dextran Sulfate Sodium (DSS). **(B)** Representative images showing the large intestines of the indicated experimental groups are shown. **(C)** Average colon length measurements are shown. **(D)** Average weight loss (relative to day 0) curves of each experimental group are shown. **(E)** Average Disease Activity Index (DAI) scores are shown. **(F–H)** CD45RB^+^ CD4^+^ T cells from WT or PANX1-KO mice were adoptively transferred into Rag2-KO mice. **(F)** Western Blots showing PANX1 expression in colon CD4^+^ T cells are shown in comparison with PANX1-KO colon CD4^+^ T cells. **(G)** Average weight loss (relative to day 0) curves of each experimental group are shown. **(H)** Average DAI scores are shown. **(A)** Data from n=10 healthy controls and n=97 UC patients. **(B–H)** Data from 2–3 independent experiments (n=6-11; western blots are representative of two independent experiments, n=4 per group). Data shown as means ± SD. *p<0.05, **p<0.01, ****p<0.0001, unpaired t-test **(A)**, One-way ANOVA + Tukey’s post-test **(C)**, multiple t-tests **(D, E, G, H)**.

In this work, our goal was to determine how PANX1-expressing CD4+ T cells (gating strategies shown on **Supplementary Figure S1**) can regulate the development of experimental colitis. We first analyzed published UC patient data (18) to trace metabolic regulators associated with UC development (**Supplementary Figure S2**). In these data we identified, among other candidates, that the gene expression of *PANX1* is preferentially expressed in UC patients compared to healthy controls (HC) (**Figure 1A**). PANX1 is a membrane-bound transporter hemichannel which is mainly known to export ATP in response to multiple stimuli or in apoptotic cells (19). Due to this correlation, we tested whether PANX1 deletion affected the development of experimental colitis. We first compared the DSS-induced colitis in wild-type versus PANX1-KO mice. We found that, in comparison to wild-type, PANX1-KO mice were significantly protected from DSS colitis, displaying increased colon length (**Figures 1B, C**), decreased weight loss (**Figure 1D**), and decreased disease (DAI) scores (**Figure 1E**, **Supplementary Figure S3A**). These results provide evidence that PANX1 expression induces the development of experimental colitis.

PANX1 is expressed by many immune and non-immune cells, including CD4^+^ T cells (20). This is also true in the context of colitis, where we found PANX1 to be expressed by colitogenic CD4^+^ T cells (**Figure 1F**). We then assessed if expression of PANX1 by effector CD4^+^ T cells is required for the induction of experimental colitis. For that, we sorted and adoptively transferred CD25^-^ CD45RB^+^ CD4^+^ T cells from wild-type or PANX1-KO mice into Rag2-KO mice, a well-established conventional CD4-induced model of colitis (21). We found that Rag2-KO mice that received PANX1-KO CD4^+^ T cells developed less colitis-associated disease, with decreased weight loss (**Figure 1G**), increased colon length (**Supplementary Figure S3B**), and decreased DAI scores (**Figure 1H**). These results suggest that expression of PANX1 by conventional CD4^+^ T cells lead to the induction of experimental colitis.

### PANX1 is required for the accumulation of GATA-3^+^ CD4^+^ T cells in experimental colitis

**Figure 2:**
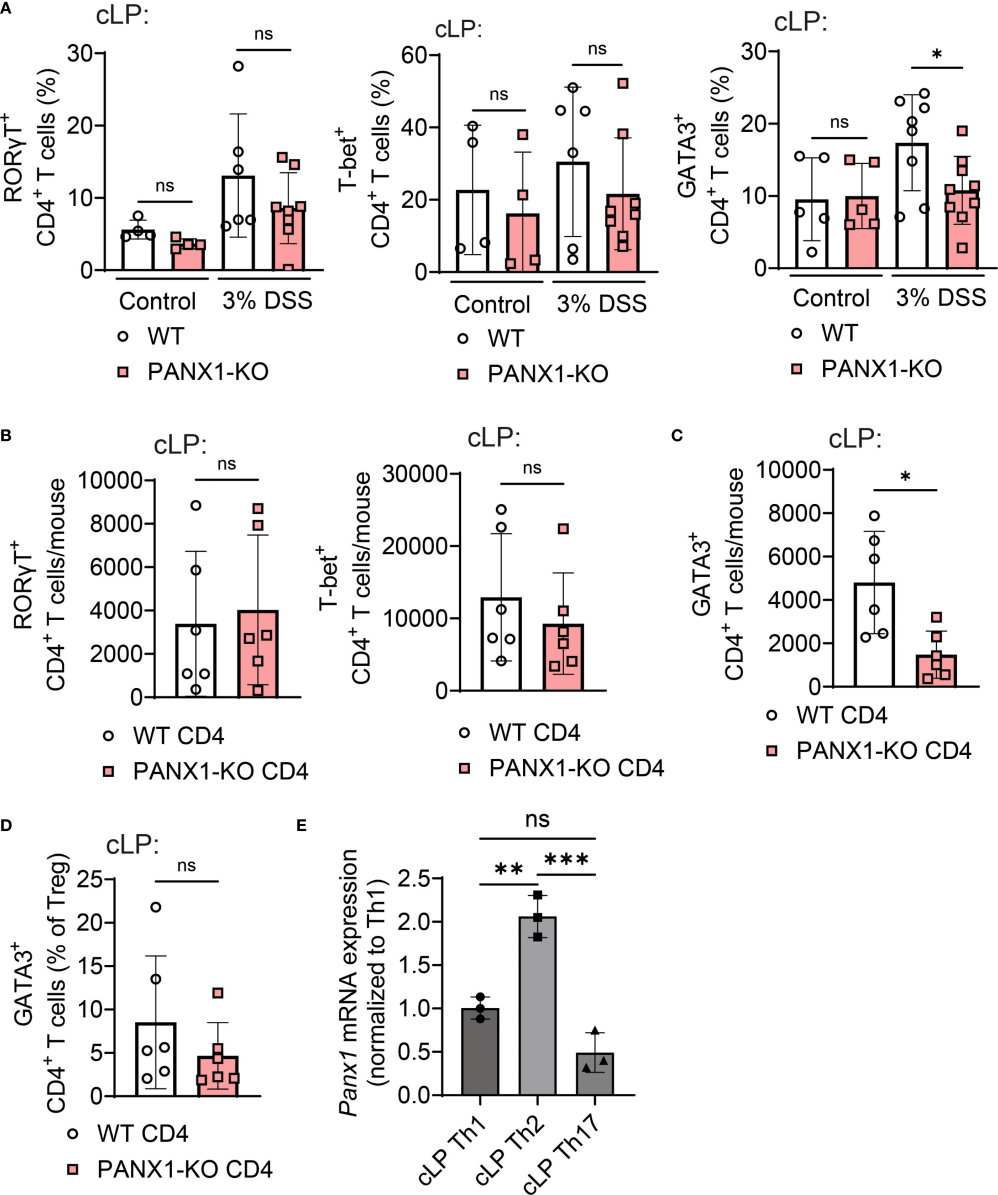
PANX1 deficiency limits the colon accumulation of GATA-3^+^ CD4^+^ T cells. **(A)** WT and PANX1-KO mice were treated between days 0–7 with PBS (control) or 3% DSS. Average cLP frequencies of RORγT^+^ CD4^+^ T cells (left) and GATA3^+^ CD4^+^ T cells (right) are shown. **(B–E)** CD45RB^+^ CD4^+^ T cells from WT or PANX1-KO mice were adoptively transferred into Rag2-KO mice. **(B)** Average cLP frequencies of RORγT^+^ CD4^+^ T cells. **(C)** Average cLP numbers of GATA3^+^ CD4^+^ T cells. **(D)** Average cLP frequencies of GATA3^+^ CD4^+^ regulatory T cells (Tregs). **(E)** *Panx1* mRNA values for T-bet^+^ Th1, GATA-3^+^ Th2 and ROR-γT^+^ Th17 cLP CD4^+^ T cells are shown. Data from 2–3 independent experiments (n=3-9). Data shown as means ± SD. *p<0.05, **p<0.01, ***p<0.001, One-way ANOVA + Tukey’s post-test.

We next assessed the colitis-induced CD4^+^ T cell immune responses altered in PANX1-KO mice. Experimental colitis is often associated with RORγT^+^ Th17 CD4^+^ T cells (7). However, we observed no differences in the colon lamina propria (cLP) numbers of Th17 CD4^+^ T cells between wild-type and PANX1-KO mice (**Figure 2A**, left). No changes in the cLP numbers of T-bet^+^ Th1 CD4^+^ T cells were detected (**Figure 2A**, middle). In contrast, we found significantly lower numbers of cLP GATA3^+^ Th2 CD4^+^ T cells in PANX1-KO mice (**Figure 2A**, right). In the CD4-induced colitis model, we found similar results, with no differences in cLP Th17 or Th1 CD4^+^ T cells, but significantly less numbers of cLP Th2 CD4^+^ T cells from PANX1-KO mice (**Figures 2B, C**). We also observed equal representation of cLP GATA3^+^ FOXP3^+^ regulatory CD4^+^ T cells (Tregs) between wild-type and PANX1-KO (**Figure 2D**), which diminishes the possibility of the effects observed in PANX1 deficiency to be due to changed accumulation of Th2-targeting induced Tregs (22). PANX1 gene expression, indeed, was higher on GATA3^+^ CD4^+^ T cells if compared to other colon-infiltrating CD4^+^ T cell populations (**Figure 2E**). Thus, PANX1 expression promotes the accumulation of colon GATA-3^+^ CD4^+^ T cells in response to experimental colitis.

### PANX1 induces TNF-α^+^ pathogenic Th2-like CD4^+^ T cells in experimental colitis

**Figure 3:**
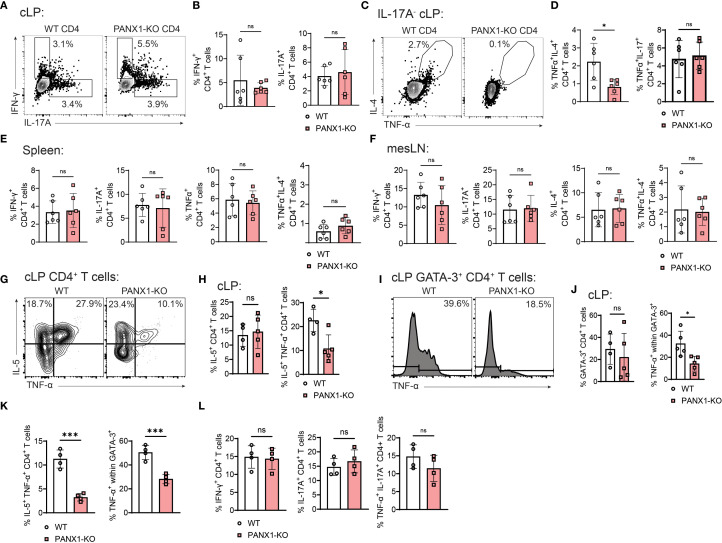
CD4^+^ T cell PANX1 induces the accumulation of TNFα^+^IL-4^+^ pathogenic Th2-like cells during colitis. **(A–F)** CD45RB^+^ CD4^+^ T cells from WT or PANX1-KO mice were adoptively transferred into Rag2-KO mice. Spleen, mesLN and cLP cells were collected and assessed for cytokine production spontaneously. **(A)** Representative flow cytometry plots showing the intracellular expression of IL-17A and/or IFN-γ by cLP CD4^+^ T cells. **(B)** Average frequencies of IFN-γ^+^ or IL-17A^+^ cLP CD4^+^ T cells. **(C)** Representative flow cytometry plots showing, within IL-17A^-^ CD4^+^ T cells, the intracellular expression of IL-4 and/or TNFα by cLP CD4^+^ T cells. **(D)** Average frequencies of TNF-α^+^IL-4^+^ cLP CD4^+^ T cells (left) and TNF-α^+^IL-17^+^ cLP CD4^+^ T cells (right). **(E)** Average frequencies of IFN-γ^+^, IL-17A^+^, TNFα^+^ and TNF-α^+^IL-4^+^ spleen CD4^+^ T cells. **(F)** Average frequencies of IFN-γ^+^, IL-17A^+^, TNFα^+^ and TNF-α^+^IL-4^+^ mesLN CD4^+^ T cells. **(G–J)** WT and PANX1-KO mice were treated between days 0–7 with PBS (control) or 3% DSS. The indicated organs were collected and assessed for cytokine production after re-stimulation ex vivo with PMA + Ionomycin **(G)** Representative flow cytometry plots showing the expression of IL-5 and TNF-α in cLP CD4^+^ T cells. **(H)** Average percentages of cLP IL-5^+^ (left) or IL-5^+^ TNF-α^+^ (right) CD4^+^ T cells. **(I)** Representative histograms showing TNF-α^+^ cLP GATA-3^+^ CD4^+^ T cells. **(J)** Average percentages of cLP GATA-3^+^ CD4^+^ T cells (left) and TNF-a^+^ within GATA-3^+^ CD4^+^ T cells (right). **(K–L)** CD45RB^+^ CD4^+^ T cells from WT or PANX1-KO mice were adoptively transferred into Rag2-KO mice. Spleen, mesLN and cLP cells were collected and assessed for cytokine production after re-stimulation ex vivo with PMA + Ionomycin. **(K)** Average percentages of cLP IL-5^+^ TNF-α^+^ CD4^+^ T cells (left) and of TNF-α^+^ within GATA-3^+^ CD4^+^ T cells (right). **(L)** Average percentages of cLP IFN-γ^+^ (left), IL-17A^+^ (middle) or TNF-α^+^ IL-17A^+^ (right) CD4^+^ T cells. Data from 2 independent experiments (n=4-6). Data shown as means ± SD. *p<0.05, ***p<0.001, unpaired t-test.

Next, we sought to further understand the phenotype of PANX1-associated colitogenic Th2 cells. We first measured the spontaneous (i.e., without re-stimulation) cytokine production pattern by wild-type versus PANX1-KO cLP CD4^+^ T cells found in Rag2-KO-transferred mice. No differences were observed between groups regarding the production of IFN-γ or IL-17A (**Figures 3A, B**), coinciding with the transcription factor data observed in **Figure 2**. In contrast, PANX1-KO CD4^+^ T cells had significantly lower proportions of TNF-α^+^IL-4^+^ cells (**Figures 3C, D**), a phenotype observed in pathogenic Th2-like CD4^+^ T cells associated with UC (8). In contrast, no differences in cLP TNF-α^+^IL-17^+^ cells were found (**Figure 3D**). These changes in cytokine production were confined to cLP CD4^+^ T cells, since no differences in cytokine production were observed in CD4^+^ T cells from spleen or mesenteric lymph nodes (**Figures 3E, F**). We found similar trends in cLP CD4^+^ T cells in WT versus PANX1-KO mice exposed to DSS, where significantly lower percentages of TNF-α^+^ Th2-like cells (IL-5^+^ GATA3^+^) upon *ex vivo* re-stimulation were found in PANX1-KO mice; in contrast, no differences in IL-5^+^ conventional Th2 cells or in other CD4^+^ T cell subsets were found (**Figures 3G–J**, **S4A, B**). Finally, we looked at the production of cytokines by CD4^+^ T cells found in Rag2-KO transferred mice upon *ex vivo* re-stimulation. In these experiments, we again found significantly reduced levels of TNF-α^+^ Th2 cells (IL-5^+^ GATA3^+^) among PANX1-KO cells, while no differences in other subsets were found (**Figures 3K, L**, **S4C**). These results suggest that the expression of PANX1 by CD4^+^ T cells promote the accumulation of TNF-α^+^ pathogenic CD4^+^ Th2-like cells in experimental colitis. Further suggesting that this effect is CD4+ T cell-specific, no differences in production of pro-inflammatory cytokines (IL-17A or TNF-α) by non-immune cells were found between WT and PANX1-KO mice exposed to DSS (**Supplementary Figure S5**).

### PANX1 promotes the Th2-like polarization and TNF-α production by CD4^+^ T cells

**Figure 4:**
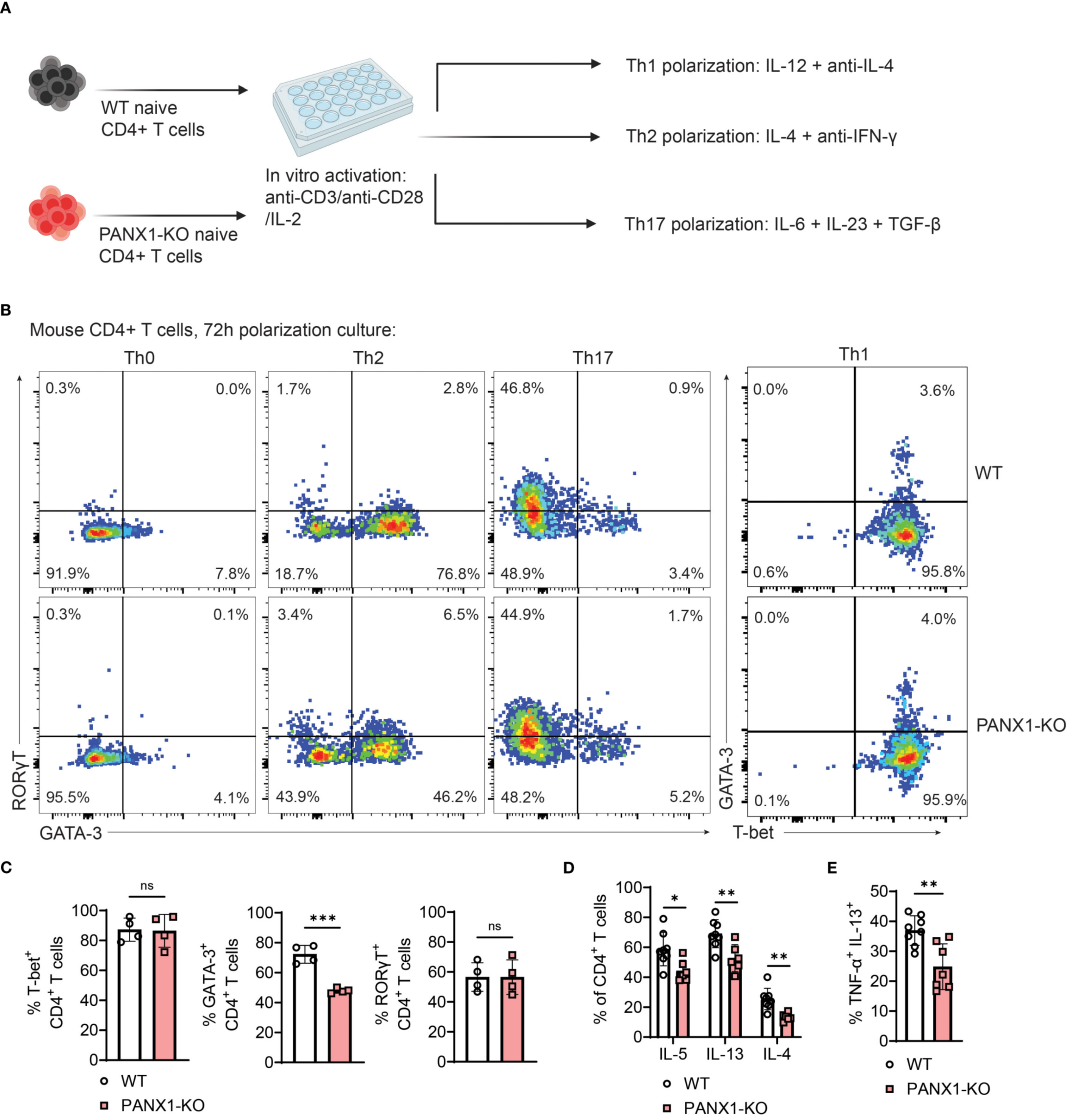
PANX1 favors the polarization of CD4^+^ T cells into TNF-producing Th2-like cells. **(A–E)** Mouse or human CD4^+^ T cells were activated *in vitro* in Th0, Th1, Th2 or Th17 polarizing conditions. **(A)** Schematics for polarization experiments. **(B)** Representative flow cytometry plots showing expression of RORγT and GATA3 in mouse WT or PANX1-KO CD4^+^ T cells *in vitro* cultured in Th0, Th2 or Th17 polarizing conditions, and for GATA-3 and T-bet expression in cells cultured in Th1 polarizing conditions. **(C)** Average percentages of T-bet^+^ CD4^+^ T cells under Th1 polarization (left), GATA-3^+^ CD4^+^ T cells under Th2 polarization (middle), and RORγT^+^ CD4^+^ T cells under Th17 polarization (right). **(D)** Average percentages of IL-5, IL-13 and IL-4 produced by cells under Th2 polarizing conditions. **(E)** Average percentages of TNF-α^+^ IL-13^+^ CD4^+^ T cells under Th2 polarizing conditions. Data from 2–3 independent experiments (n=4–8 per group). Data shown as means ± SD. *p<0.05, **p<0.01, ***p<0.001, unpaired t-test.

We then assessed the cell-intrinsic role of PANX1 in the induction of the Th2 effector phenotype in CD4^+^ T cells. For this, we *in vitro* activated wild-type or PANX1-KO naïve CD4^+^ T cells in multiple distinct polarizing conditions (**Figure 4A**). PANX1 was dispensable for the effector polarization into Th1 or Th17. In contrast, a significant reduction in Th2 cell polarization was observed in PANX1-KO CD4^+^ T cells, as evidenced by both reduced levels of GATA3 and of type 2 cytokines such as IL-5, IL-13 and IL-4 (**Figures 4B–D**). Importantly, we also observed reduced percentages of *in vitro* Th2 cells with the ability to produce TNF-α in PANX1-KO (**Figure 4E**). These results provide additional evidence that PANX1 is important for the induction of the Th2 cell effector and TNF-α production by Th2-phenotype CD4^+^ T cells, mirroring our *ex vivo* findings (**Figures 2**, **3**).

### CD4^+^ T cell PANX1 induces the accumulation of neutrophils during experimental colitis

**Figure 5:**
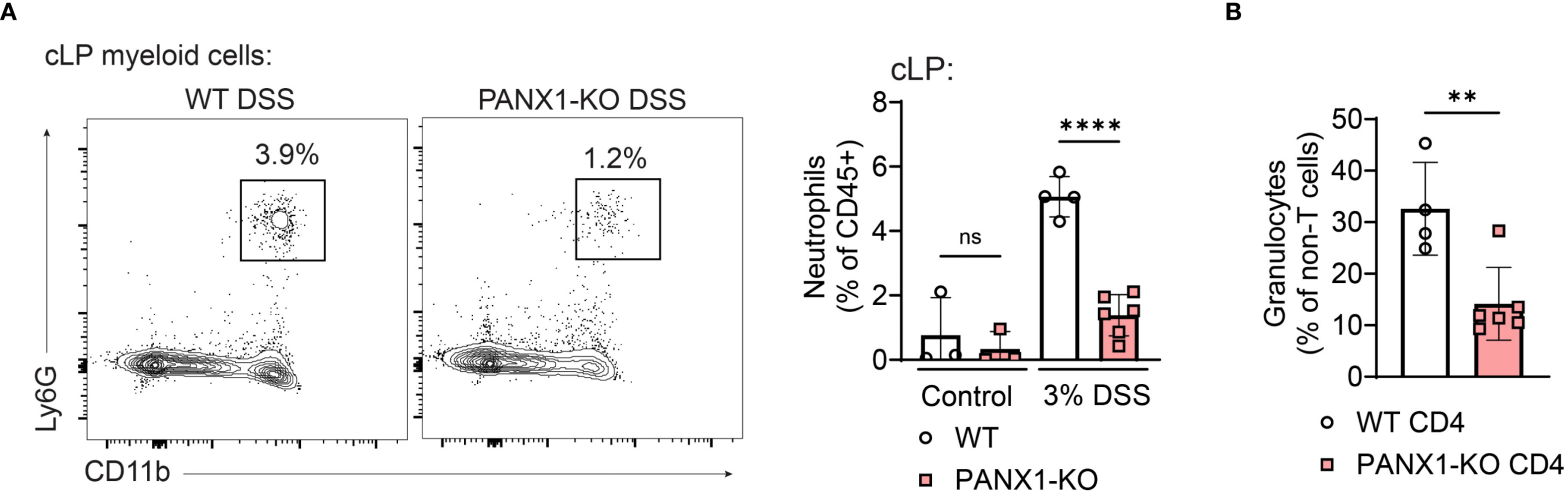
PANX1 deficiency decreases the colon accumulation of neutrophils following experimental colitis. **(A)** WT and PANX1-KO mice were treated with PBS (control) or 3% DSS. In the left, representative flow cytometry plots showing expression of CD11b and Ly6G in cLP myeloid cells (CD3^-^); CD11b^+^Ly6G^+^ neutrophils are depicted in the gates shown. In the right, average percentages (within CD45^+^ cells) of cLP neutrophils in the indicated groups are shown. **(B)** CD45RB^+^ CD4^+^ T cells from WT or PANX1-KO mice were adoptively transferred into Rag2-KO mice. Average percentages of cLP SSC^high^CD11b^+^ granulocytes (within CD3^-^ cells) are shown. Data from 2 independent experiments (n=3-6). Data shown as means ± SD. **p<0.01, ****p<0.0001, One-way ANOVA + Tukey’s post-test **(A)**, unpaired t-test **(B)**.

Finally, we tracked which innate immune cells are driven by PANX1-dependent CD4^+^ Th2 cells. In wild-type versus PANX1-KO mice with DSS-induced colitis, we observed no differences in the colon accumulation of macrophages, monocytes, eosinophils or dendritic cells (**Supplementary Figure S6A**). In contrast, DSS-treated PANX1-KO mice displayed decreased proportions of colon neutrophils (**Figures 5A**, **S6B**). Likewise, in Rag2-KO mice transferred with PANX1-KO CD4^+^ T cells, we observed decreased accumulation of granulocytes (**Figures 5B**, **S6B**). Altogether, these data show that, during experimental colitis, CD4^+^ T cell expression of PANX1 leads to the colon accumulation of neutrophils. These data also suggest that colitogenic neutrophilia is likely dependent on PANX1 CD4^+^ T cell expression and is positively correlated with pathogenic Th2 CD4^+^ T cell responses. Indeed, UC patient samples had enriched gene signatures associated with pathogenic Th2-like cell responses (23) and colitogenic neutrophilic responses (24) (**Supplementary Figure S7**).

## Discussion

The progression of colitis depends, among other factors, on the accumulation and effector function of pathogenic CD4^+^ T cell responses (3). However, there is still incomplete knowledge about the metabolic regulators driving colitogenic CD4^+^ T cell differentiation, especially cell-intrinsic regulators such as transmembrane metabolite transporters. In this report, we identified that one of these transporters, PANX1, plays an instrumental role in the formation of pathogenic CD4^+^ T cells and their induction of experimental colitis. We found that PANX1 expression promotes the accumulation of TNF-α^+^ pathogenic Th2-like CD4^+^ T cells, which correlated with increased colitis disease and host weight loss. We also found a correlation between PANX1-dependent accumulation of colitogenic Th2 cells and the accumulation of neutrophils in colitis mice. Altogether, our data suggests that, beyond reported functions in enteric nervous system cells (16), PANX1 serves as a cell-intrinsic regulator of pathogenic CD4^+^ T cell responses driving colitis.

PANX1 is a membrane transporter whose main known function is the export of ATP to the extracellular milieu (19). PANX1 has known physiological roles in the induction of apoptotic cell clearance (25) and blood pressure control (26). In addition, its importance as a regulator of T cell responses has been increasingly appreciated, with reported roles for the induction of T cell activation *in vitro* (27) and *in vivo* CD8^+^ T cell responses to infection and tumors (15). More recently, PANX1 was shown to be protective against lung allergic inflammation through an increase in Treg function and inhibition of pathogenic CD4^+^ T cells (14). In contrast with this report, our findings show a pro-disease function of PANX1 in experimental colitis through induction of pathogenic CD4^+^ T cell responses. This dichotomy between pathogenic CD4^+^ T cells in the lungs versus the colon may have many possible explanations. We did not observe differences in Treg numbers in PANX1-KO mice, and the protective effect of PANX1-KO is carried over to Rag2-KO hosts receiving only conventional CD4^+^ T cells, making it unlikely thymically-derived Tregs are involved in the PANX1-KO mediated phenotype. Tregs are important in the regulation of intestinal inflammation (28, 29), although some reports suggest a correlation between the presence of colon Tregs and increased colitis (30). A possible role for PANX1-expressing peripherally induced Tregs cannot be discarded and should be tested in future research, despite no observed differences in Treg numbers in Rag2-KO hosts receiving WT or PANX1-KO conventional CD4^+^ T cells.

We found that, instead of an effect on Th17 cells, which are widely known to contribute to colitis (7), PANX1 promoted the accumulation of TNF-α^+^ Th2-like, pathogenic cells. TNF-α is not commonly produced at high levels by Th2 cells (9), but its release by colitogenic Th2-like cells has been previously reported (8). The signals driving the accumulation of these pathogenic Th2-like cells, however, are not well understood. Our data suggests a cell-intrinsic role for PANX1 in their generation, as well as in the induction of the Th2 cell fate. Given PANX1’s major role in exporting ATP (19), the channel could influence Th2 cell fate through two potential mechanisms. First, PANX1-mediated accumulation of extracellular ATP (eATP) could act in an autocrine and/or paracrine way by activating P2X receptors such as P2RX7 (31), which are expressed by CD4^+^ T cells, including Th2 cells (32). Alternatively, PANX1-induced changes in intracellular ATP levels could drive changes in metabolic pathways such as the AMPK pathway in a cell-intrinsic manner (15). AMPK overactivation can, indeed, drive increased proliferation and TNF-α production by CD4^+^ T cells (33). However, AMPK has been suggested to hinder Th2 responses in mouse models of lung allergy (34) and colitis (35), making this less likely to be the pathway for PANX1-mediated induction of colitogenic CD4^+^ T cell responses. Future studies will be necessary to identify the role of eATP accumulation versus the contribution of intracellular changes in ATP, as this was a limitation of our current work. Another gap in our study is the lack of functional assessments of PANX1 channels in Th2-like cells. We did observe increased expression of *Panx1* mRNA by cLP Th2-like cells, but whether this is accompanied by increased PANX1 channel function remains to be determined.

Unexpectedly, the PANX1-mediated effect in pathogenic Th2 cell responses was correlated with increased colon neutrophil accumulation. Neutrophilia is a common inflammatory driver in UC (24), but neutrophil accumulation is often linked to pathogenic Th17 responses (36), which are not altered in a PANX1-dependent way. It is still possible that PANX1-dependent changes in neutrophil accumulation occur due to Th17 responses in a tissue-compartmentalized way that cannot be captured by flow cytometry-based approaches. Nevertheless, TNF-α plays a crucial role in the recruitment of neutrophils during colitis (37), suggesting that the potential connection between PANX1, colitogenic Th2-like cells, and neutrophilia occur through TNF-α production and release in the colon.

In summary, we found that expression of the ATP-exporting hemichannel PANX1 by CD4^+^ T cells significantly promote colitis inflammation, a phenotype linked to the accumulation of pathogenic, TNF-producing Th2-like cells and neutrophils. We also found that PANX1 can drive the differentiation of TNF-producing Th2-like cells *in vitro*. Future mechanistic studies will be necessary to define whether and how ATP export via PANX1 drive the formation of pathogenic Th2-like cells, and how these cells can influence the accumulation of neutrophils during colitis. Overall, our findings provide important evidence for the role of transmembrane metabolic regulators in the formation of pathogenic immune responses during colitis, a mechanism that may be true in other pathogenic scenarios.

## Methods

### Analyses of human UC genomic data

RNA-seq data from the clinical studies deposited as the GSE75214 (ref.18) were downloaded and analyzed using the R-Studio software for differentially expressed genes between healthy controls and UC patients. Differentially expressed genes were considered when the FDR adjusted p-value was under 0.05 (adj.p <0.05) and logFC values were greater than 0.5.

### Mice

Male and female mice, aged 6–8 weeks, with a specific-pathogen-free status, were used in this study. Mice were housed in the animal facilities of The Department of Immunology at Mayo Clinic Arizona. All mice were randomly assigned to experimental groups. The following mouse strains were utilized: C57BL/6, CMV-Cre (WT), CMV-Cre *Panx1* ^fl/fl^ (PANX1-KO), Rag2-KO. For *in vivo* experiments, mice were sacrificed using CO_2_ inhalation (fill rate of 30-70% displacement of the chamber volume per minute with CO_2_). All experimental procedures were conducted in accordance with institutional guidelines and were approved by the institutional Animal Care and Use Committee (IACUC - A00005542-20-R23).

### DSS-mediated induction of colitis

WT or PANX1-KO mice were treated with 3% Dextran Sulfate Sodium (DSS) diluted in water for the induction of colitis. These mice were monitored for up to 10 days for weight loss and disease activity index (DAI, described in **Supplementary Figure S2**). At the endpoints, mice were sacrificed for analyses of colon length and flow cytometry.

### CD45RB^+^ CD4^+^ T cell adoptive transfer colitis experiments

For adoptive transfer-mediated colitis experiments, splenocytes were isolated from WT and PANX1-KO mice. CD45RB^+^CD25^-^CD4^+^ T cells were isolated using cell sorting and transferred intravenously into RAG2-KO mice (4x10^5^ cells per mice). Mice were monitored for up to 8 weeks for weight loss and DAI. At the endpoints, mice were sacrificed for analyses of colon length and flow cytometry.

### Tissue processing for flow cytometry

At the endpoints, the colons, mesenteric lymph nodes (mLNs) and spleens were collected. Colons were processed and digested with Dithioerythritol (DTE, 0.5 mg/ml, Sigma-Aldrich) for 30 min at 37°C for collection of cIEL cells and with collagenase type IV (0.5 mg/mL) (Gibco) at 37 °C for 40 minutes under agitation (200 rpm) for collection of cLP cells. Mesenteric lymph nodes (mLNs) and spleens were mechanically processed using cell strainers (Corning). The cell suspension obtained from the tissues was homogenized, filtered through cell filters, and washed with PBS containing 10% FBS. Afterward, the cell suspension was centrifuged at 1,200 rpm for 5 minutes and resuspended in FACS buffer until the time of staining.

### Flow cytometry analysis

Cell suspensions from the cIEL, cLP, mLNs and spleen were stained with fluorochrome-conjugated monoclonal antibodies (indicated throughout the text), diluted in FACS buffer and incubated at 4°C for 40 minutes. After staining, cells were washed with FACS buffer and then prepared for intracellular cytokine and transcription factor staining. For this, cells were incubated with Cell Stimulation Cocktail Plus Protein Transport Inhibitors (Invitrogen - 2 μM) for 4 hours at 37 °C in 5% CO_2_. After incubation, cells were fixed and permeabilized using the Cytofix/Cytoperm kit (BD Biosciences) or the Transcription Factor Staining kit (eBioscience) and then washed with the buffer provided in the kits. A Live/Dead dye (Cytek Tonbo) was used to identify dead cells. Samples were analyzed using multiparametric flow cytometry with a FACS Symphony (BD Biosciences) flow cytometer. Data was analyzed using FlowJo software (BD Biosciences).

### CD4^+^ T cell polarization *in vitro*

Spleen CD4^+^ T cells from WT or PANX1-KO mice were isolated using the EasySep Naïve CD4^+^ T cell isolation kit (StemCell Biotechnologies). CD4^+^ T cells were then activated *in vitro* with 10 μg/ml anti-CD3 and 1 μg/ml of anti-CD28, in the presence of 10 ng/ml mouse recombinant IL-2. For Th1 polarization, cell cultures were supplemented with recombinant IL-12 (10 ng/ml) and anti-IL-4 (10 ng/ml); for Th2 polarization, cell cultures were supplemented with recombinant IL-4 (10 ng/ml) and 10 μg/ml of anti-IFN-γ; for Th17 polarization, cell cultures were supplemented with recombinant TGF-β (2 ng/ml), recombinant IL-6 (50 ng/ml), 10 ug/ml of anti-IFN-γ and 5 ug/ml of anti-IL-4. After 48h, CD4^+^ T cells were assessed for polarization by flow cytometry.

### Western blot

Western Blots were done based on our previous study (15). Briefly, the indicated CD4^+^ T cells were lysed in RIPA buffer (+ 1 mM PMSF and protease/phosphatase inhibitors). Cell lysates were sonicated and protein concentrations measured through BCA assays. Protein aliquots (25 μg) were run on 4-12% agarose gradient gels and transferred to nitrocellulose membranes (Trans-Blot Turbo system). Membranes were blocked for 30 minutes at room temperature with TBS, then stained with primary rabbit anti-Pannexin-1 (Cell Signaling Technologies, 1/1000) or mouse anti-Tubulin (Millipore, 1/3000), at 4°C overnight. After washing, secondary staining with anti-rabbit or anti-mouse antibodies were done (1/15000 dilution, 1h room temperature). After washing, membranes were imaged using a LICOR Odyssey DLx system.

### Quantitative PCR analyses

Quantitative PCR analyses were done as previously described (38), using triplicates for each sample. We used amplification kits detected with ROX Sybr Green Master Mix (Applied Biosystems), using the QuantStudio 7 Pro sequence detection system. For analysis, we used the delta-delta Ct algorithm. Cycling threshold values for the control target gene (*Actb*) were subtracted from cycle threshold values for *Panx1*. We used the following primers in this study: *Panx1*-FW: GTGGCTGCACAAGTTCTTCCC; *Panx1*-RV: GATGGCGCGGTTGTAGACTT; *Actb*-FW: TGAGCTGCGTTTTACACCCT; *Actb*-RV: TTTGGGGGATGTTTGCTCCA.

### Statistical analysis

Specific statistical tests applied to each experiment are detailed in the respective figure legends. All analyses were conducted using GraphPad Prism 10 software. Data are presented as Mean values with standard deviation (SD) shown as error bars. For comparisons between two groups, Paired or Unpaired T-tests were used. Differences between groups were considered significant when p < 0.05 (*), p < 0.01 (**), p < 0.001 (***) or p<0.0001 (****).

## Data Availability

Publicly available datasets were analyzed in this study. This data can be found here: GSE75214.
