# Supplementary material for: Pannexin-1 hemichannels promote experimental colitis inflammation in a CD4+ T cell-specific manner
Source: Front Immunol. 2025 Oct 8;16:1621353. doi: 10.3389/fimmu.2025.1621353 (PMC12540062; doi:10.3389/fimmu.2025.1621353)
Supplement: Supplementary file 1 [file DataSheet1.pdf]

Supplementary Data

Title: Pannexin-1 hemichannels promote experimental colitis inflammation in a CD4^{+} T cell-specific way

Authors: Pooja Rani Mina[1], Bruna de Gois Macedo[1], Caio Loureiro Salgado[1], Chloe Liliana Leff[1], Daniel Bihnam[1], Angad Beniwal[1,2], Igor Santiago-Carvalho[1], Nhan Le Tran[1,2], Henrique Borges da Silva[1,2#]

1 – Department of Immunology, Mayo Clinic, Phoenix, AZ, United States

2 – Department of Cancer Biology, Mayo Clinic, Phoenix, AZ, United States

#Corresponding author and Lead Contact:

Henrique Borges da Silva

Department of Immunology, Mayo Clinic

5777 E Mayo Blvd, IERB 2-306, Phoenix, AZ, United States, 85054

Phone: +1 480-301-3592

E-mail: borgesdasilva.henrique@mayo.edu

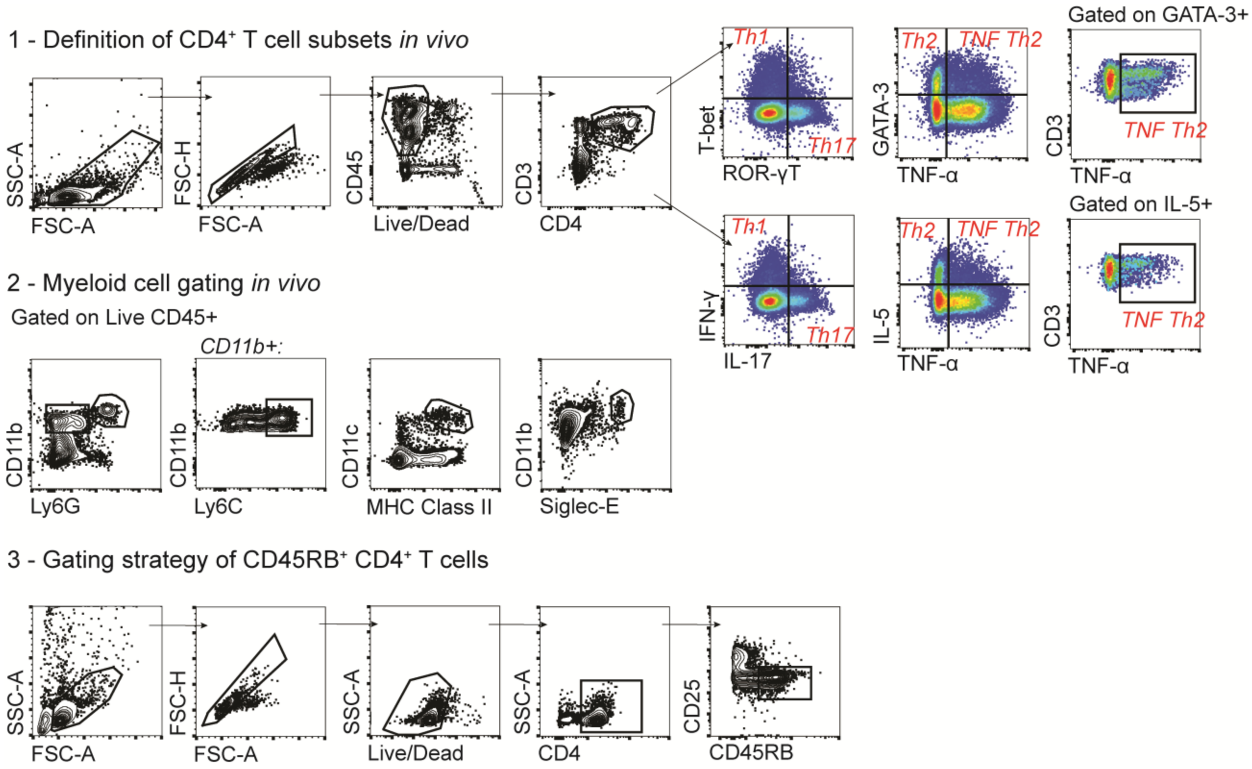

Figure S1. Gating strategies for flow cytometry experiments. Gating strategies for the definition of CD4+ T cell subsets in vivo (1), myeloid cell gating in vivo (2), and for the identification of CD45RB+ CD4+ T cells prior to sorting (3) are shown.

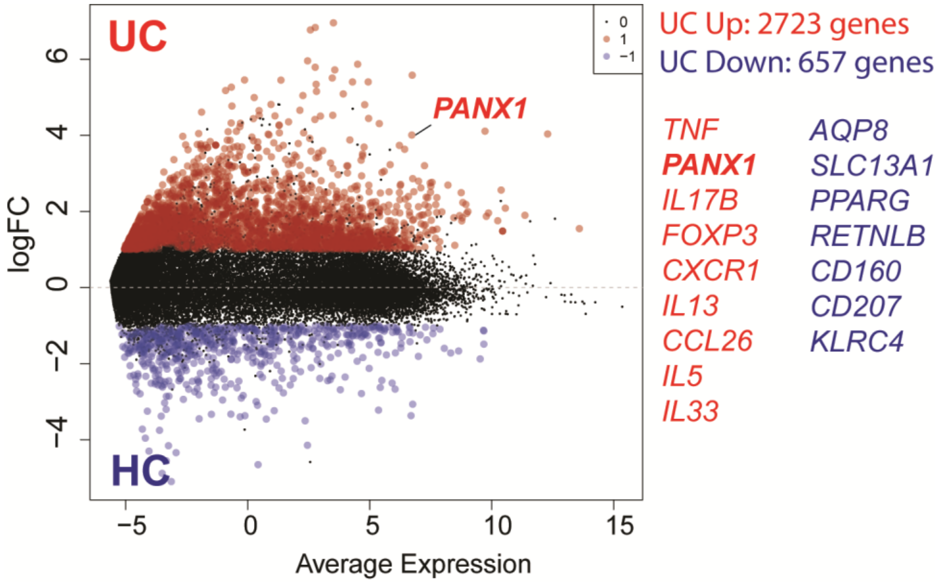

Figure S2. UC-associated transcriptional signatures. Differential mRNA expression of genes in UC (depicted in red) versus HC (depicted in blue) donors, data from ref.18. Data from n=10 healthy controls and n=97 UC patients. Significant differences in mRNA expression were considered when logFC >0.5 (or <-0.5) and adjusted p<0.05.

A

**Disease Activity Index**										
The fecal blood, stool consistency, and body weight of mice will be monitored. DAI will be examined by the scores of										
**1-** Fecal blood (0, no observed blood; 1, a small amount of blood in some stool; 2, blood in stool regularly seen; and 3, blood in all stools)										
**2-** Stool consistency (0, regular; 1, soft but still formed; 2, very soft; and 3, diarrhea),										
**3-** Body weight loss (0, no loss; 1, 1–5% loss; 2, 5–10% loss; and 3, 10– 20% loss)										
DAI=1+2+3										

B

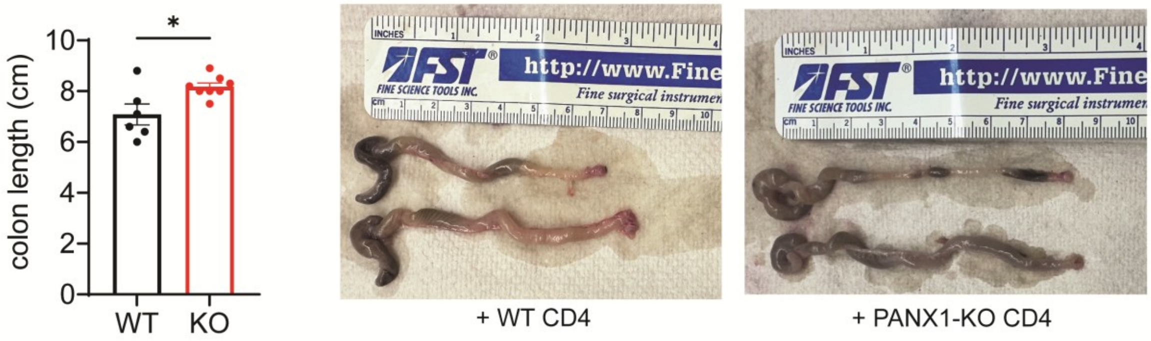

Figure S3. Effects of CD4+ T cell PANX1 deletion in a mouse model of colitis. (**A**) Criteria used for disease index scores (DAI) measurements. (**B**) CD45RB+ CD4+ T cells from WT or PANX1-KO mice were adoptively transferred into Rag2-KO mice. Average colon length values (left) and representative images showing the large intestines in the experimental groups (right) are shown. Data from 3 independent experiments (n=6-8). Data shown as means ± SD. *p<0.05, unpaired t-test.

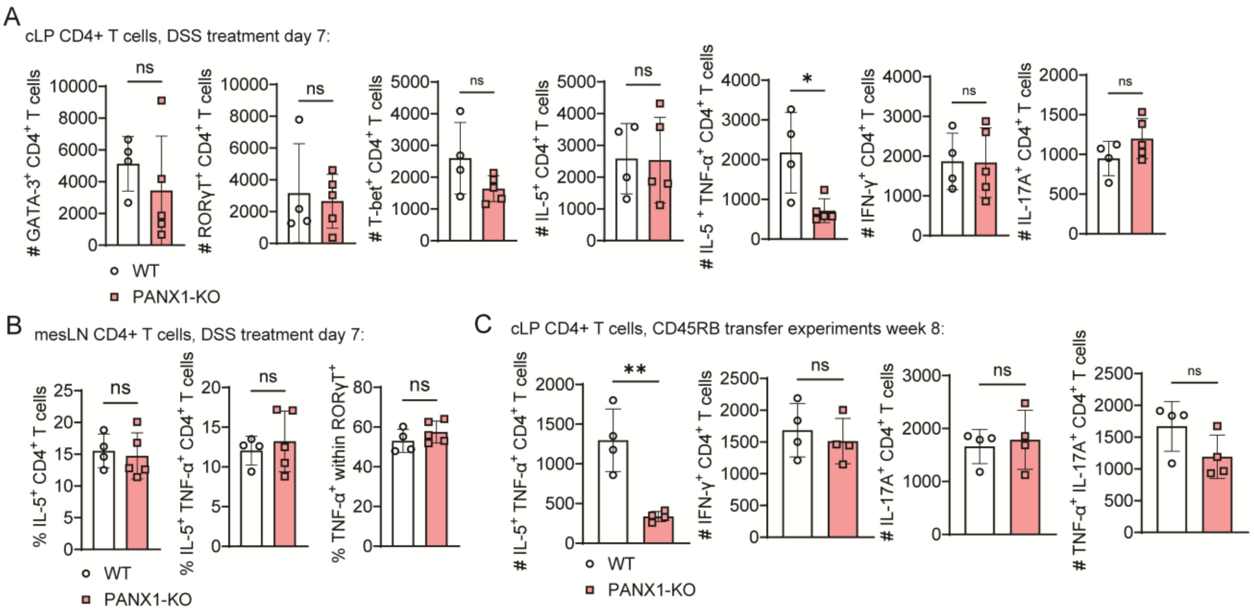

Figure S4. PANX1 promotes the numerical accumulation of IL-5^{+} TNF-α^{+} pathogenic Th2 CD4^{+} T cells in the colon in response to experimental colitis. (**A-B**) WT and PANX1-KO mice were treated between days 0-7 with PBS (control) or 3% Dextran Sulfate Sodium (DSS). Cells were assessed after re-stimulation ex vivo with PMA + Ionomycin. (**A**) Average numbers of the indicated populations of cLP CD4^{+} T cells are shown. (**B**) Average percentages of the indicated populations of mesLN CD4^{+} T cells are shown. (**C**) CD45RB^{+} CD4^{+} T cells from WT or PANX1-KO mice were adoptively transferred into Rag2-KO mice. Cells were assessed after re-stimulation ex vivo with PMA + Ionomycin. Average numbers of the indicated populations of cLP CD4^{+} T cells are shown. Data from 2 independent experiments (n=4-5). Data shown as means ± SD, Unpaired-test.

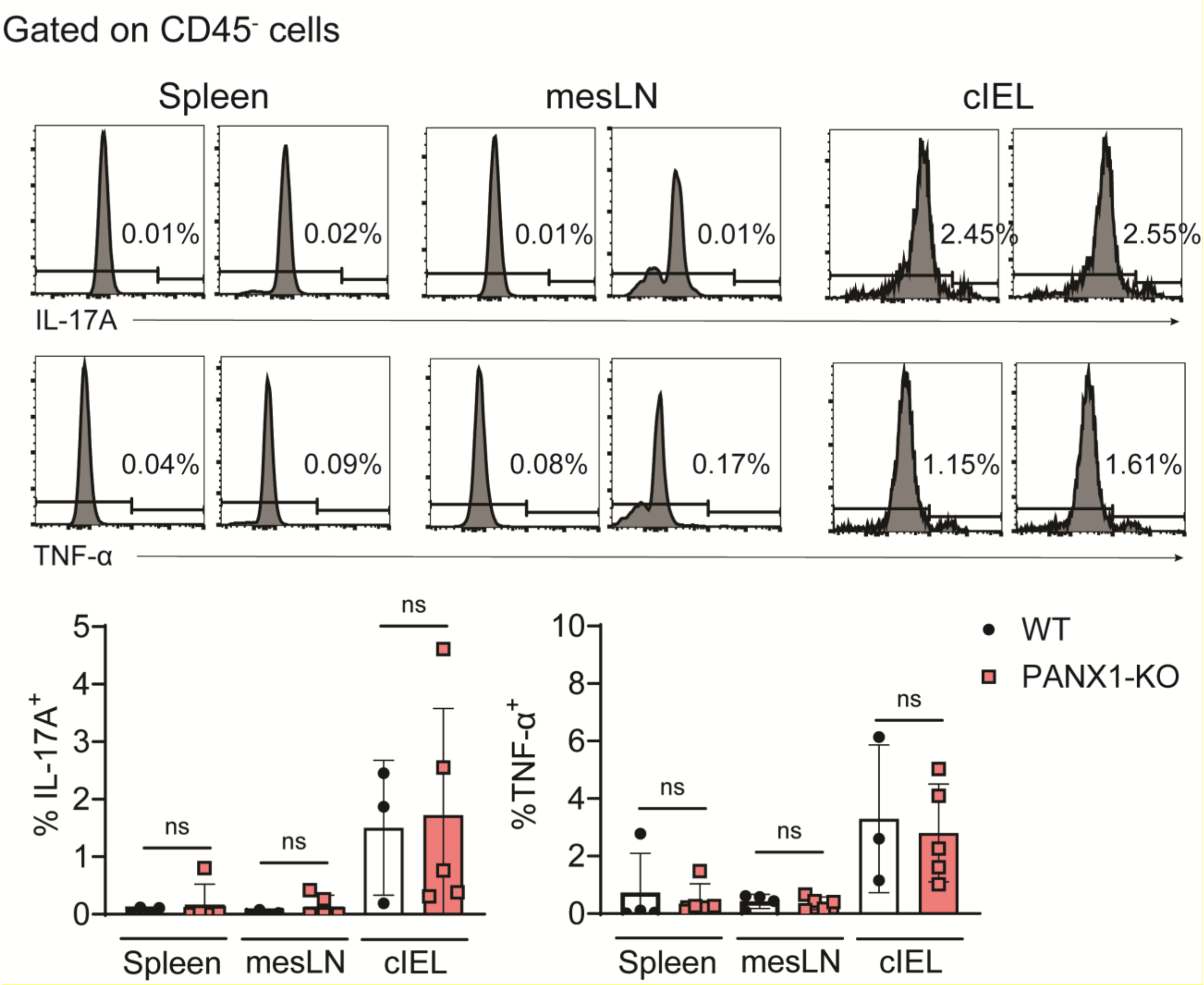

Figure S5. PANX1 does not play a role in the production of pro-inflammatory cytokines by non-immune cells in response to DSS colitis. WT and PANX1-KO mice were treated between days 0-7 with PBS (control) or 3% DSS. Cells were assessed after re-stimulation ex vivo with PMA + Ionomycin. Representative histograms (top) and average percentages (bottom) of IL-17A and TNF-α by spleen, mesLN or cIEL $CD45^-$ non-immune cells are shown. Data from 2 independent experiments, n=4-5. Data shown as means ± SD, One-way ANOVA + Tukey's post-test.

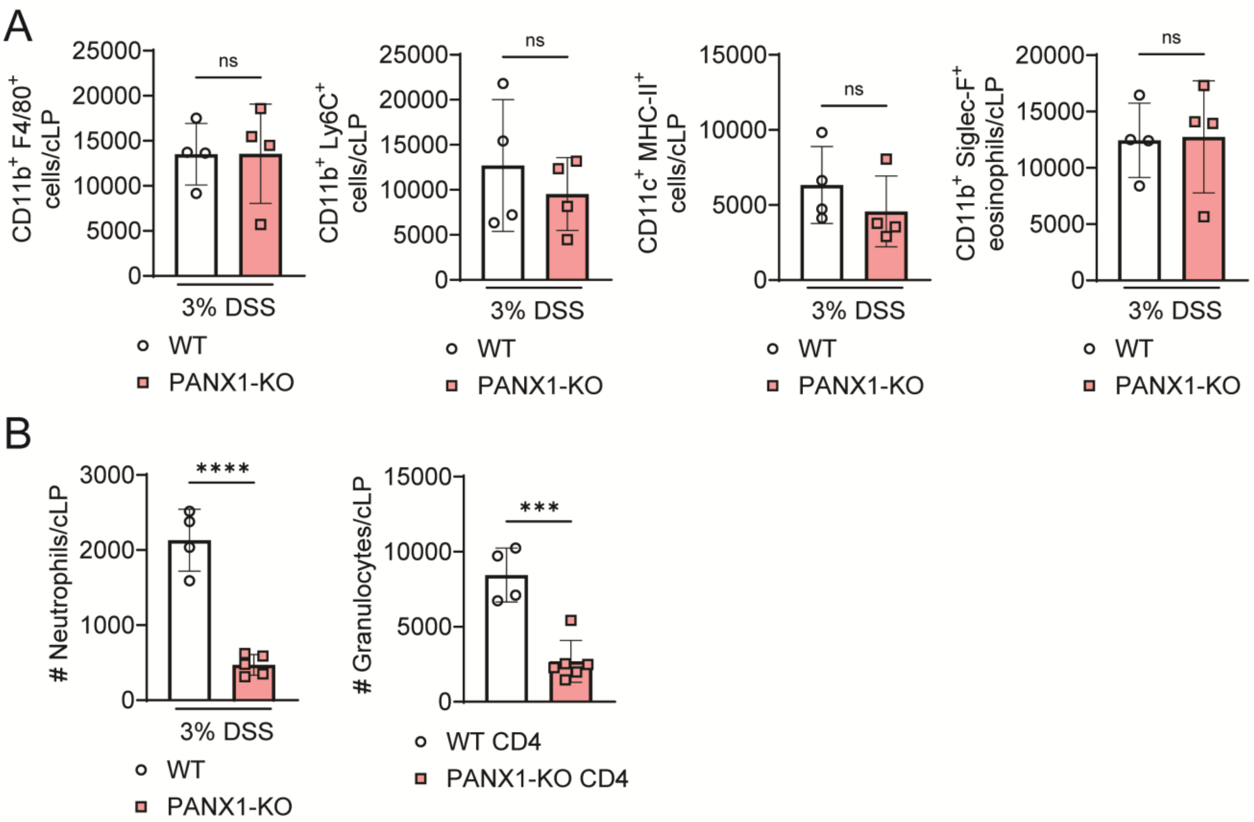

Figure S6. In contrast with granulocytes, PANX1 is dispensable for the colon accumulation of macrophages, monocytes, dendritic cells or eosinophils during experimental colitis. (A) WT and PANX1-KO mice were treated between days 0-7 with PBS (control) or 3% Dextran Sulfate Sodium (DSS). Average numbers of cLP CD11b$^+$F4/80$^+$ macrophages, CD11b$^+$Ly6C$^+$ inflammatory monocytes, CD11c$^+$MHC-II$^+$ dendritic cells, and CD11b$^+$Siglec-F$^+$ eosinophils are shown. (**B**) Average numbers of cLP neutrophils from DSS-treated mice (left) and average numbers of cLP granulocytes (SSC^{hi}) from Rag2-KO mice transferred with CD45RB$^+$ CD4$^+$ T cells (right) are shown. Data from 2-3 independent experiments (n=4-6). Data shown as means ± SD (Unpaired t-tests made).

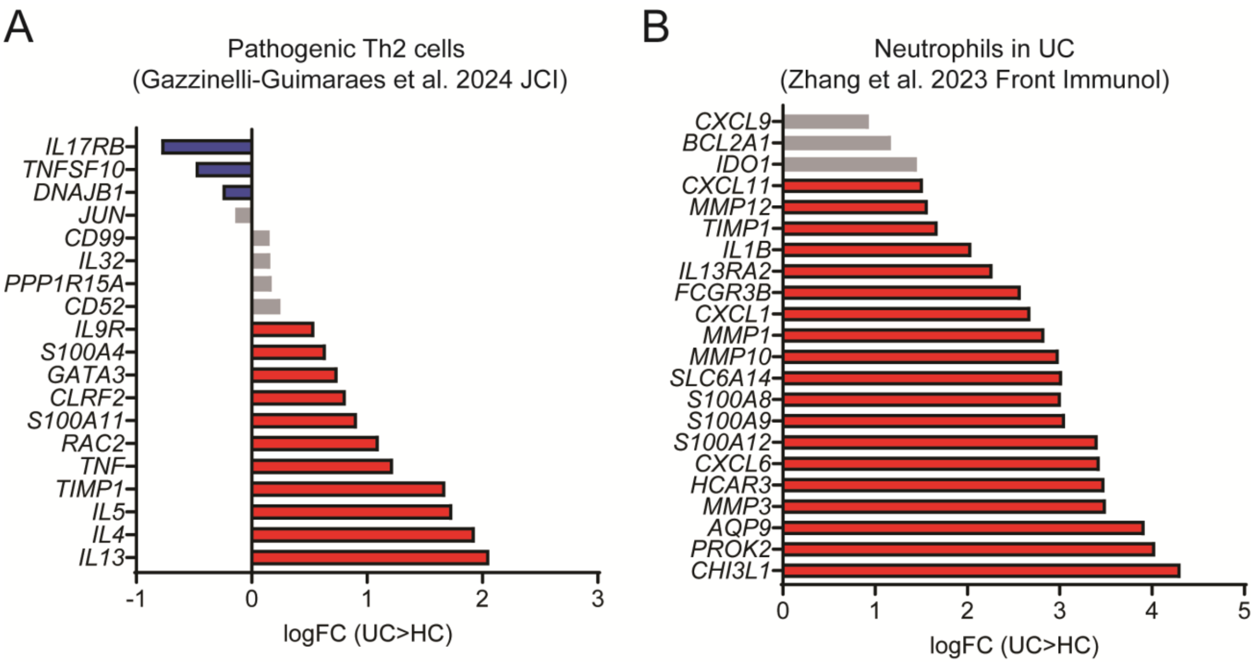

Figure S7. UC-associated transcription correlates with pathogenic Th2 and neutrophilia signatures. (**A-B**) Using the logFC values from the differentially expressed genes (adj.p<0.05) from the data of Figure S1, we tracked the expression of genes listed to be associated with distinct UC-associated pathogenic signatures as defined by ref. 23 and 24, namely pathogenic Th2 cells and neutrophilia. In red, genes significantly upregulated in UC patients. In blue, genes significantly decreased in UC patients. (**A**) UC/HC logFC values of genes associated with pathogenic Th2 cells as listed by ref.23. (**B**) UC/HC logFC values of genes associated with neutrophilia in UC as listed by ref.24.
